# Current trends in the features of male thyroid cancer: Retrospective evaluation of their prognostic value

**DOI:** 10.1097/MD.0000000000015559

**Published:** 2019-05-13

**Authors:** Min Jhi Kim, Seul Gi Lee, Kwangsoon Kim, Cho Rok Lee, Sang-Wook Kang, Jandee Lee, Kee-Hyun Nam, Woong Youn Chung, Jong Ju Jeong

**Affiliations:** a Department of Surgery, Samsung Medical Center, Sungkyunkwan University School of Medicine; b Department of Surgery, Severance Hospital, Yonsei Cancer Center, Yonsei University College of Medicine, Seoul, South Korea.

**Keywords:** male sex, oncologic outcome, papillary microcarcinoma, papillary thyroid cancer, sex disparity

## Abstract

This study aimed to compare the clinicopathologic characteristics and oncologic outcomes of papillary thyroid cancer (PTC) patients according to sex. Then, we validated prognostic variables to determine whether sex is a significant prognostic factor for PTC.

Between January 2007 and December 2010, 1232 men and 7276 women PTC patients underwent surgery. The patient characteristics and 5-year oncologic outcomes were compared. The stepwise Cox proportional hazards model determined the significance of survival variables.

Men PTC patients had more lymph node (LN) metastases than women, both in the total (*P* < .0001) and hemithyroidectomy (*P* < .0001) patients. Men and women showed similar 5-year recurrence-free survivals, both in total (*P* = .815) and hemithyroidectomy (*P* = .148) patients. The tumor size and the number of positive central nodes were associated with tumor recurrence, but not sex.

Sex was not an independent prognostic factor for tumor recurrence. Multicenter clinical studies with long-term follow-ups are needed to validate these results.

## 1 Introduction

Sex is an important factor related to the pathogenesis, diagnosis, and prognosis of many diseases.^[1]^ Sex disparities have been discovered in various cancers, and can easily be observed in sex-specific organs such as the breast, ovary, uterus, and prostate. However, differences in the incidence and/or prognosis are also shown in some sex-shared sites, including the thyroid, lung, and skin.^[2,3]^

The thyroid cancer incidence has increased worldwide over the last decade, making it one of the most common endocrine tumors. It accounts for 1% of all cancers worldwide,^[4]^ with a 6.6% annual percent change in the United States from 1997 to 2009.^[5]^ The increased incidence is predominantly observed for tumors with papillary histology, with no significant changes seen in cases of follicular, medullary, or anaplastic histology. The increase includes mainly papillary microcarcinomas (PMCs), defined as tumors <1 cm, although the incidence of large tumors has also increased.^[6,7]^

With the rising incidence, thyroid cancer is currently the seventh most common malignancy in women; however, it is not among the most common 15 cancers in men. The 2002 GLOBOCAN database reported 103,589 female thyroid cancer cases worldwide, compared with 37,424 male cases, representing a female-to-male ratio of >2:1.^[8]^ This sex disparity appears dominantly in differentiated thyroid cancer (DTC), such as papillary thyroid cancer (PTC) and follicular thyroid cancer (FTC), the incidence of which is 3-times higher in women than in men.^[9]^

Patients with thyroid cancer have a favorable prognosis, with the 10-year survival generally reported to exceed 90%. However, multiple studies have reported that men have a worse prognosis than women.^[10–12]^ From the analysis of large retrospective uncontrolled studies, sex has been identified as a significant prognostic factor for thyroid cancer along with other parameters such as age, tumor size, histologic type, multicentricity, extrathyroidal extension, and lymph node (LN) metastases.^[10,12]^ Among the several staging classifications for thyroid cancer, the Age, Metastases, Extent and Size and the European Organization for the Research and Treatment of Cancer criteria include sex as a variable.^[13,14]^

Considering the sex differences in the incidence patterns, aggressiveness, and prognosis of thyroid cancer, the present study aimed to first identify and compare recent trends in clinicopathologic characteristics and oncologic outcomes of both male and female PTC patients. Second, we aimed to validate prognostic variables to determine whether sex is a significant prognostic indicator for PTC.

## 2 Methods

### 2.1 Patients

To discover recent trends of PTC, medical records of patients who were diagnosed with PTC from January 2007 to December 2010 were reviewed. A total of 8508 PTC patients, including 1232 male patients (14.5%) and 7276 female patients (85.5%), underwent thyroid surgery at the Department of Surgery in Yonsei University College of Medicine. Among them, hemithyroidectomy was performed for 250 male and 1501 female patients. Initially, comparative analyses were performed for all patients, followed by subgroup analyses among the hemithyroidectomy patients, according to sex. All procedures performed in this study were in accordance with the ethical standards of the Institutional Review Board of Yonsei University Health System (4–2016–0538) and with the 1964 Declaration of Helsinki and its later amendments or comparable ethical standards.

### 2.2 Preoperative work-up

The preoperative patient work-up included physical examination, high-resolution staging ultrasonography (US), and neck computed tomography (CT). Thyroid nodules were diagnosed preoperatively based on US-guided fine needle aspiration biopsy (FNAB). In patients with DTC, the size and location of tumor, degree of extrathyroidal extension, nodal involvement, and other abnormal findings in the neck were complementarily evaluated by preoperative US and neck CT.^[15]^ If a patient had clinically palpable LNs or a LN with a suspicious appearance on preoperative US, FNAB was performed for the LN. The presence of LN metastasis was determined by ultrasonography-guided FNAB or by the thyroglobulin (Tg) levels in the FNAB washout fluid (FNA-Tg >10 ng/mL, >the mean + 2SD of the FNA-Tg measured in node-negative patients, or >serum-Tg) from the LN.^[16]^ Meanwhile, LN micrometastases were defined as metastatic deposits within a LN of <2 mm in diameter, which are rarely detected by US (clinical N0).^[17]^

### 2.3 Surgery

The criteria for the extent of surgery were determined in accordance with the American Thyroid Association guidelines.^[18]^ Hemithyroidectomy was indicated if the following conditions were satisfied: patient age <45 years, a single lesion <1 cm in size, no definite extrathyroidal invasion, no LN metastasis on preoperative imaging studies, no personal history of the disease in the head or neck, and no first-degree family history of DTC. In all other cases, total thyroidectomy was indicated.

Prophylactic ipsilateral central compartment neck dissection (CCND) was conducted in all patients. The central compartment LNs consist of the level 6 LNs (pretracheal, prelaryngeal, and paraesophageal LNs), which include the LNs from the hyoid bone superior to the suprasternal notch inferiorly. On each side, the lateral boundary is limited by the medial border of the carotid sheath. For patients diagnosed with metastatic thyroid cancer in the lateral neck, neck dissection of levels IIa, III, IV, and VB was performed. The surgery followed the approaches of modified radical neck dissection type III for PTC, sparing the sternocleidomastoid muscle, spinal accessory nerve, and internal jugular vein. The LNs at levels IIB and VA were not routinely dissected.^[19,20]^

All patients were administered with levothyroxine for thyroid-stimulating hormone (TSH) suppression immediately postoperatively. Radioactive iodine (RAI) ablation was performed based on each patient's stage and risk factors, according to American Thyroid Association guidelines, at 4 to 12 weeks after surgery.^[18]^ These patients underwent post-therapy whole-body scans and diagnostic whole-body scans 2 to 5 days after RAI ablation, and abnormal RAI uptakes were investigated.^[21]^

### 2.4 Outcome assessment

To evaluate oncologic outcomes, the TSH-suppressed serum Tg concentration was measured annually, and all patients underwent regular follow-up US. Patients with evidence of recurrence or distant metastasis were assessed using other imaging modalities, such as neck CT and/or positron emission tomography-CT. Regional neck node recurrence was confirmed by US-guided FNAB.

Overall survival (OS) was defined as the time from the date of initial surgery to the date of the last follow-up or death. Recurrence-free survival (RFS) was defined as the time from the surgery to the date of detection of the first recurrence on imaging.

### 2.5 Statistical analysis

Categorical data are reported as the rates and proportions, while the median and ranges were calculated for continuous data. Differences in continuous variables between men and women were compared using the Mann–Whitney *U* test or the Student *t* test, and differences in categorical variables were compared with the chi-square test or Fisher exact test, as appropriate. Patient characteristics and 5-year oncologic outcomes were compared in the 2 sex groups.

Survival curves were analyzed using the Kaplan–Meier method, and statistical significance was determined using the log-rank test. Significance of survival variables was analyzed by the stepwise Cox proportional hazards model. Univariate Cox regression analysis determined the correlation between each variable and RFS. Multivariate Cox regression analysis assessed whether sex was independently associated with RFS. *P*-values <.05 were considered statistically significant. All data were processed and statistically analyzed using IBM SPSS Statistics for Windows, version 23.0. (IBM Corp., Armonk, NY).

## 3 Results

### 3.1 Patient characteristics

Table 1 shows the clinicopathologic characteristics of the patients according to sex. Male patients underwent more total thyroidectomies with CCND and lateral neck dissection than women (16.4 vs 11.2%; *P* < .001). The median tumor size was larger (0.8 [0.5–1.2] vs 0.7 [0.5–1.1] cm; *P* < .001), and the median numbers of retrieved central (4 [2–7] vs 5 [3–8]; *P* = .001) and lateral neck nodes (29 [20–46] vs 23 [8–33]; *P* < .001) were higher in men. Furthermore, male patients had greater numbers of positive central (1 [0–2] vs 0 [0–1]; *P* < .001) and lateral neck nodes (4 [1–8] vs 2 [0–5]; *P* < .001), which resulted in significantly more frequent N1a and N1b stage tumors in male patients (49.7 vs 33.9%; *P* < .001). After surgery, larger number of male patients received RAI treatment (58.0 vs 54.8; *P* = .032). Higher median RAI dose was applied to male patients (30 [0–30] vs 30 [0–30] mCi; *P* < .001) (Table 1).

**Table 1:**
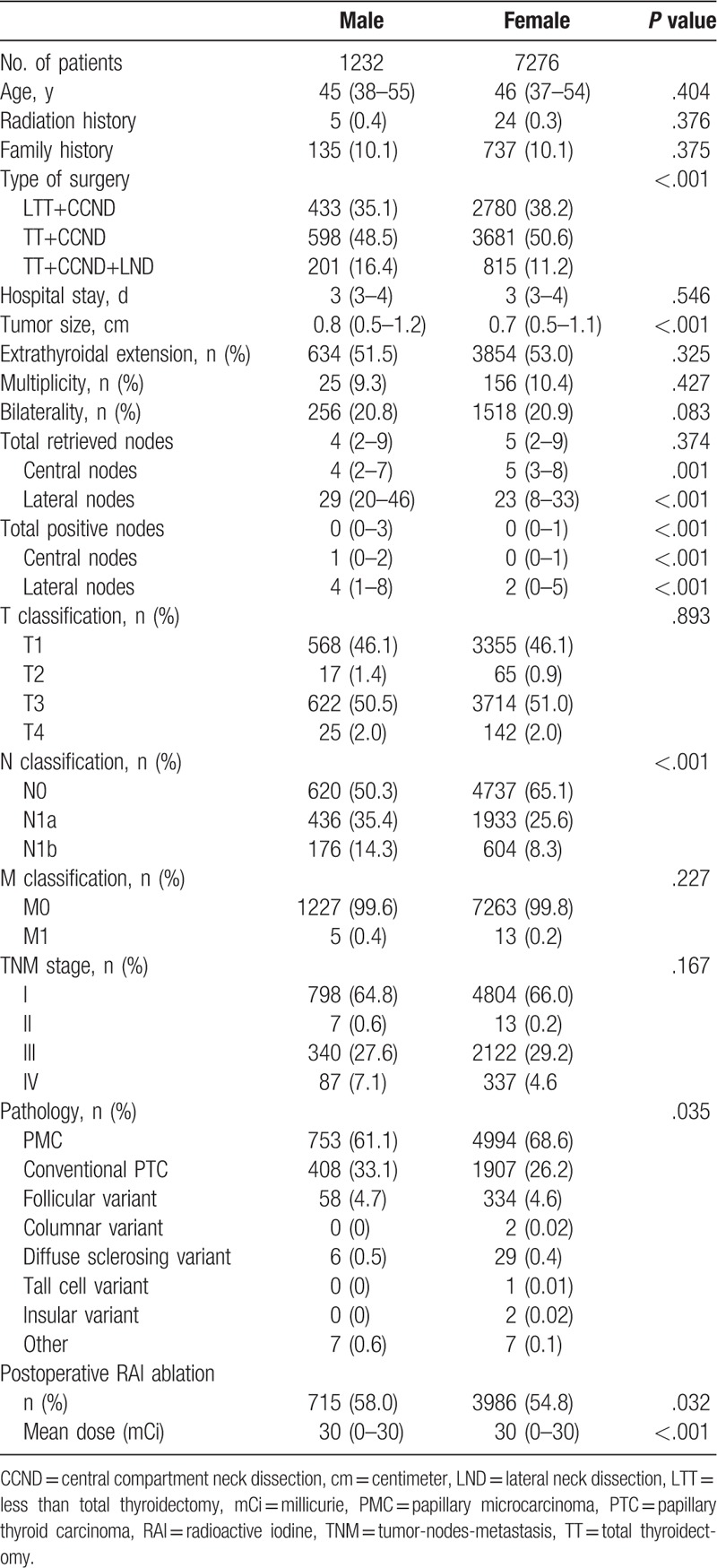
Clinicopathologic characteristics of study patients according to sex.

The median follow-up duration was 58 (range, 50–102) months. One hundred five male patients and 392 female patients were lost to follow-up.

### 3.2 Oncologic outcomes

The ablation success rates based on diagnostic whole-body scans were similar in the matched groups (95.7 vs 95.6%; *P* = .746). The TSH-suppressed serum Tg concentrations at 5 years after ablation were also similar (3.2 ± 13.6 vs 3.7 ± 24.9 ng/mL; *P* = .593). During the follow-up, 21 male and 87 female patients experienced tumor recurrence. The recurrence rates over 5 years were not significantly different between the 2 groups (1.7% vs 1.0%; *P* = .531). Patients with recurrence showed similar clinicopathologic features, recurrence-free period, site of recurrence, treatment modality, and serum Tg concentration at 5 years (Table 2). There was no disease-specific mortality during the follow-up period.

**Table 2:**
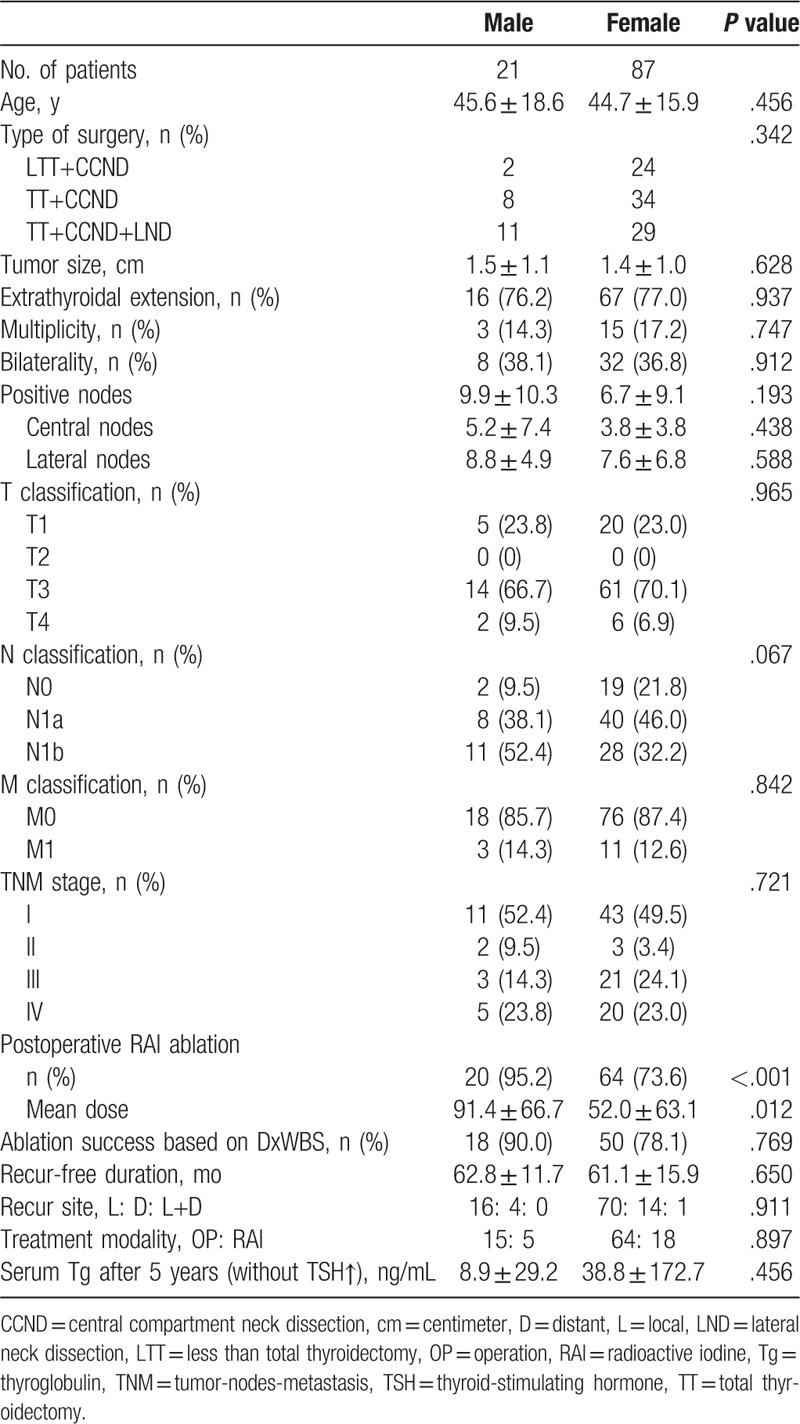
Analysis of patients with tumor recurrence according to sex.

**Figure 1:**
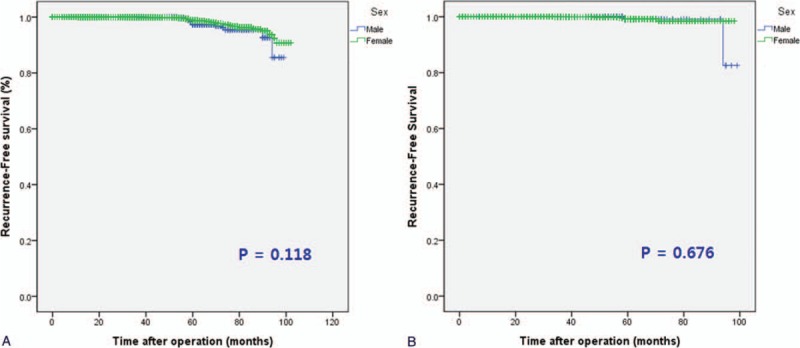
Kaplan–Meier estimates of recurrence-free survival in (A) all patients and (B) hemithyroidectomy patients according to sex.

The 5-year median RFS did not differ significantly between the 2 groups (55.7 ± 17.8 vs 59.0 ± 17.9 months; *P* = .118) (Fig. 1A).

### 3.3 Recurrence-free survival (RFS) analysis

Univariate survival analyses were performed to determine the correlations between sex and other clinicopathological characteristics and RFS. No difference in RFS was found between male and female patients (hazard ratio [HR] 1.529, 95% confidence interval [CI] 0.949–2.463). Subsequently, multiple Cox survival analyses were performed for RFS to show that mean tumor size was associated with RFS. Sex was not an independent predictor of RFS (Table 3).

**Table 3:**
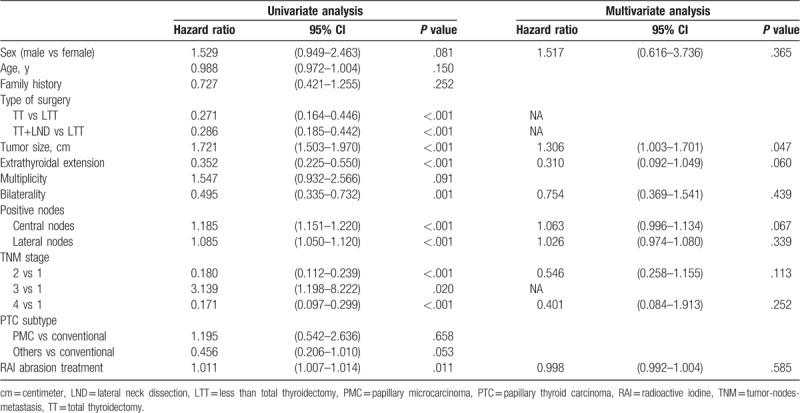
Stepwise Cox regression analysis of PTC patients.

### 3.4 Subanalyses of patients treated with hemithyroidectomy

Further assessment of the PTC patients who underwent hemithyroidectomy from January 2007 to December 2010 was conducted. As a result, male patients were found to be older (44 [37–52] vs 42 [35–50] years; *P* = .007). The median number of retrieved central neck nodes was higher in female patients (3 [2–6] vs 4 [2–7]; *P* = .002), whereas male patients had a greater number of positive central neck nodes (0 [0–1] vs 0 [0–0]; *P* < .001), and consequently more frequent N1a stage tumors (30.4 vs 19.0%; *P* < .001) (Table 4).

**Table 4:**
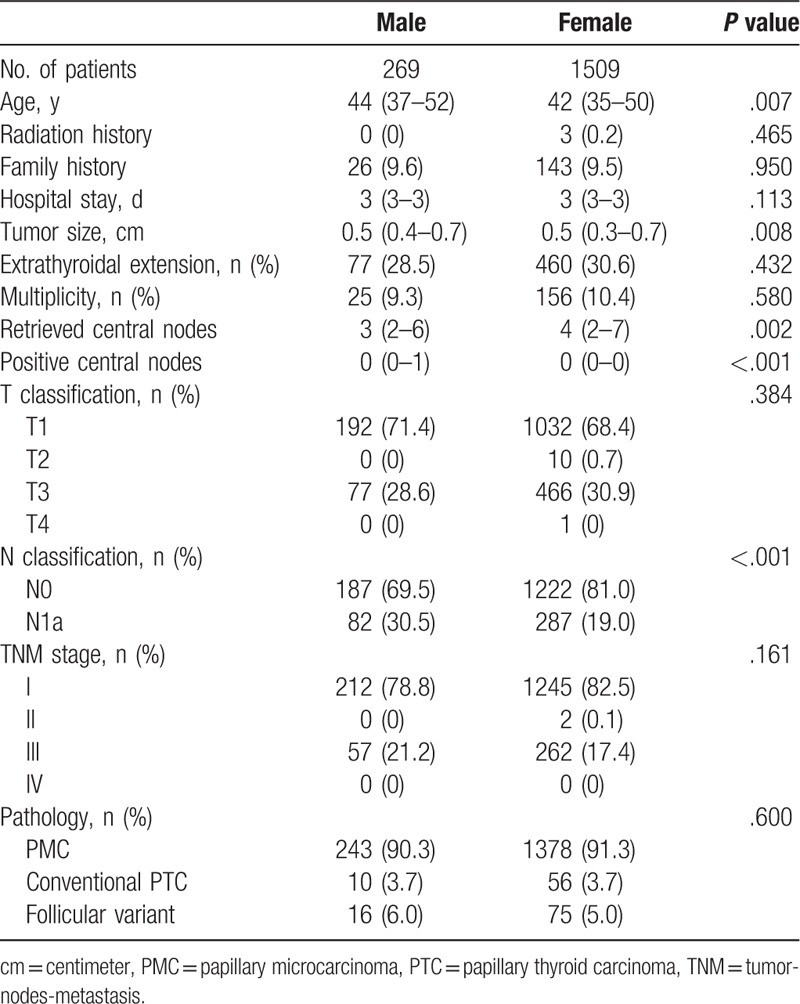
Clinicopathologic characteristics of hemithyroidectomy patients according to sex.

Recurrence was detected in 2 male and 8 female patients after 5 years. The recurrence rates were similar in the 2 groups (0.7 vs 0.5%; *P* = .672). Patients with recurrence had similar clinicopathologic features, postoperative RAI treatment, recurrence-free period, site of recurrence, treatment modality, and serum Tg concentration after 5 years (Table 5).

**Table 5:**
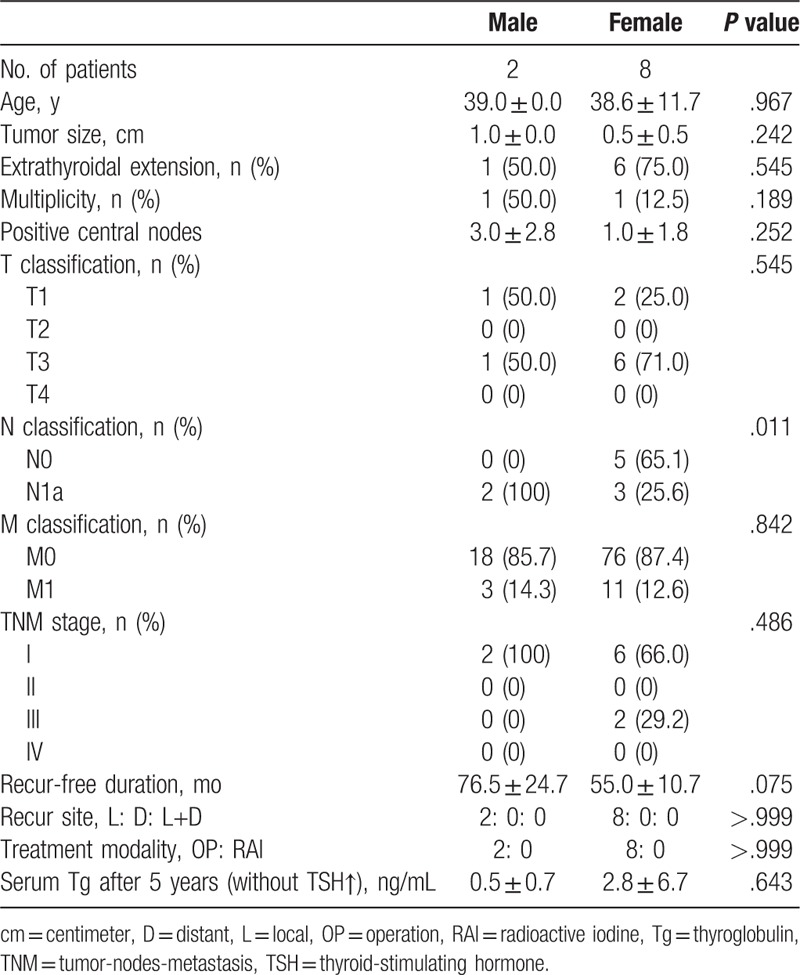
Analysis of hemithyroidectomy patients with tumor recurrence according to sex.

The 5-year median RFS showed no significant difference between the 2 groups (54.2 ± 18.5 vs 66.0 ± 16.9 months; *P* = .676) (Fig. 1B).

Univariate and multiple Cox regression analyses were repeated for hemithyroidectomy patients. Univariate analysis revealed no difference in RFS between male and female patients (HR 1.327, 95% CI 0.278–6.323). Multivariate Cox survival analyses showed that the number of positive central neck nodes was an independent predictor of RFS, whereas sex was not an independent predictor of RFS (Table 6).

**Table 6:**
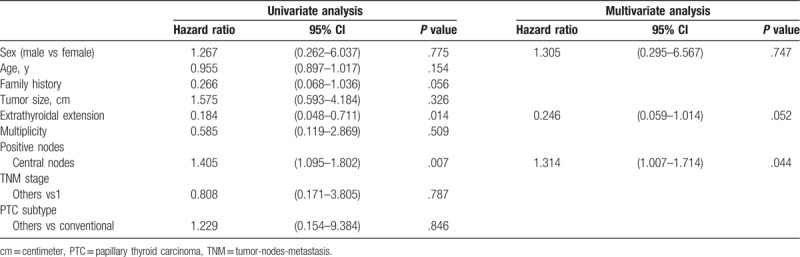
Stepwise Cox regression analysis of hemithyroidectomy patients.

To investigate the appropriate cutoff tumor size that yields similar nodal status in hemithyroidectomy patients, the chi-square test was performed for sex and N stage in all final pathologic tumor sizes. A tumor size ≤0.25 cm was significantly associated with the similar nodal status between the 2 sex groups.

Moreover, we compared pathological N1a (pN1a) patients who received hemithyroidectomy. Eighty-two male and 161 female patients were analyzed and showed similar rates of LN micrometastases according to preoperative staging US (79.3 vs 81.7%; *P* = .962). US also revealed similar sizes of suspicious LNs in the 2 groups (0.5 ± 0.2 vs 0.6 ± 0.2 cm; *P* = .203).

## 4 Discussion

Thyroid cancer is one of the most common endocrine malignancies; it shows notable sex disparities, with a higher incidence in women and worse prognosis in men.^[8,10–12,22]^ The sex difference in incidence is remarkable in DTCs, such as PTC and FTC, for which the incidence is 3 times higher in women than in men.

PTC is the most common histologic type, accounting for approximately 80% of cases. Its incidence has nearly doubled in the last few decades, contributing to the remarkable increase in the overall thyroid cancer incidence worldwide.^[9]^ While this may be due to the earlier diagnosis of subclinical disease,^[23]^ little is currently known about the factors responsible for the dramatic increase in PTC.

Investigations regarding the causes of these sex disparities have been conducted previously. Radiation exposure^[24]^ and dietary iodine,^[25]^ well-known environmental risk factors for thyroid cancer, do not appear to account for these disparities. Clinical and epidemiologic studies have provided no consistent evidence regarding common somatic genetic alterations such as BRAF V600E, that are rearranged in transformation/papillary thyroid carcinomas, or neurotrophin receptor tyrosine kinase 1 mutations contributing to the thyroid cancer sex differences.^[26,27]^ Recent studies have suggested that the estrogen and estrogen hormone receptor status in thyroid cancer cells may promote cell proliferation in thyroid cancer; however, there is no conclusive evidence to confirm the correlation between sex hormones and these disparities.^[28,29]^

Male sex was associated with a higher mortality and lower disease-free survival in most previous studies, and men show worse OS and disease-specific survival (DSS) with more recurrence.^[30–39]^ Meanwhile, other studies have shown no sex differences, presenting with the unaffected OS and DSS.^[30,36,40–42]^ Thus, a male survival disadvantage is not a consistent finding, and the impact of sex on recurrences of DTC is not yet substantiated.

Herein, we analyzed and compared the clinicopathologic characteristics and oncologic outcomes of male and female PTC patients. The study period included PTC patients from 2007 to 2010 for 2 reasons: to involve cases representative of the recent trends in PTC, and to assess the 5-year oncologic outcomes. Moreover, the stepwise Cox proportional hazards model was created to evaluate significant survival variables. First, univariate Cox regression analysis identified the correlations between each variable and RFS. Second, multivariate Cox regression analysis was performed to discover whether sex was independently associated with RFS.

Our analyses revealed that male PTC patients had significantly more LN metastases than females, both in the central and lateral neck areas. However, despite the different nodal statuses, the 2 groups showed similar 5-year median RFS. Sex was not an independent prognostic factor for tumor recurrence. According to our data, tumor size was associated with prognosis of PTC, and the number of positive central nodes had prognostic capacity in hemithyroidectomy patients.

Tumor size was traditionally considered a prognostic factor for disease recurrence, and the extent of surgery as well as RAI treatment after surgery is largely determined by tumor size. PTCs were generally divided into PMC and conventional PTC on the basis of a tumor diameter of 1.0 cm. However, the previous studies have reported controversial recurrence rates according to tumor size cutoff of 1.0 cm. Recent studies provide the optimal tumor size cutoff of 1.8 to 2.0 cm for predicting the risk of recurrence.^[43,44]^ Moreover, many international groups have also revealed the prognostic significance of increasing number and ratio of positive LNs in DTC.^[45–47]^ These findings are comparable to our study.

Although the reason for the similar RFS between male and female PTC patients shown in this study remains difficult to explain, it may be partially due to the long-term trend of decreasing prognostic potential of male sex. One study has reported secular trends in the prognostic factors for PTC. The risk for tumor recurrence associated with male sex decreased over time, while the risk associated with pathologic characteristics either remained the same or increased. Such results suggest that the reasons behind this trend may be the change in clinical characteristic of PTC and increased early detection of cancer in male patients.^[48]^

In hemithyroidectomy patients, those with a tumor size of ≤0.25 cm were found to have similar N stages. Small-sized PMCs rarely metastasize to the LNs, both in men and women. Thus, early-stage thyroid cancers such as tiny PMC may not show considerable sex difference in LN involvement. We furthermore found that the 82 male and 161 female pN1a hemithyroidectomy patients had similar rates of LN micrometastases and similar sizes of suspicious LNs. Male PTC patients may have more metastatic potential to the LNs than females; however, the metastatic LNs themselves may show similar cancer behaviors and traits, especially in early-stage thyroid cancers.

This study has several limitations. First, the analysis was based on a limited group of patients. Even with the existing indications of total thyroidectomy and hemithyroidectomy, the early days of thyroid surgery tend to be rather aggressive. In the early- and mid-2000s, total thyroidectomy was predominantly performed in thyroid cancer patients. Hence, our comparative analyses involved patients treated from 2007, during which the number of less than total thyroidectomies gradually increased. Second, the study population was from a single center. Our institution offers a well-developed screening program for thyroid cancer patients, and a substantial amount of early-stage PTCs may have been included. Moreover, we routinely perform prophylactic ipsilateral CCND to all thyroid cancer patients. Results of this study should be interpreted while considering these backgrounds. Third, as PTC has a relatively favorable prognosis and mild biological behavior, long-term follow-up data on the oncologic outcomes are needed to validate our results.

Despite the aforementioned limitations, the present study is the first to compare the oncologic outcomes of PTC patients according to sex. We believe that our study has provided some novel viewpoints on current trends in male PTC patient prognosis. The overall increasing incidence of PMC that suggests prompt diagnosis and treatment in the recent era should alert clinicians to carefully consider the disease characteristics themselves, not just sex differences. This study suggests that aggressive pathologic factors have more impact on disease prognosis than sex. The prognostic indicators for PTC, including large tumor size or LN metastases, should be focused on to provide proper treatment. Based on past statistical data, male sex linked to more aggressive treatment can possibly be a fallacy of generalization. Our results provide a good starting point to guide future studies.

## 5 Conclusion

Despite the higher metastatic potential for the LNs in male PTC patients, sex was not an independent prognostic factor for tumor recurrence. The similar oncologic outcomes and the similar traits of metastatic LNs among men and women indicate that excessive treatments in male PTC patients compared with females may not be warranted, especially in cases of PMC. Multicenter clinical studies with long-term follow-ups are needed to validate these results.

## Author contributions

**Conceptualization:** Min Jhi Kim, Jong Ju Jeong.

**Data curation:** Min Jhi Kim, Seul Gi Lee, Kwangsoon Kim, Cho Rok Lee, Sang-Wook Kang, Jandee Lee, Kee-Hyun Nam, Woong Youn Chung, Jong Ju Jeong.

**Formal analysis:** Jong Ju Jeong.

**Funding acquisition:** Jong Ju Jeong.

**Investigation:** Min Jhi Kim.

**Methodology:** Min Jhi Kim, Jong Ju Jeong.

**Project administration:** Min Jhi Kim, Jong Ju Jeong.

**Resources:** Min Jhi Kim.

**Supervision:** Jong Ju Jeong.

**Validation:** Min Jhi Kim.

**Writing – original draft:** Min Jhi Kim.

**Writing – review & editing:** Min Jhi Kim.

Min Jhi Kim orcid: 0000-0002-7791-2994.
